# Comparison between completely and traditionally retroperitoneoscopic nephroureterectomy for upper tract urothelial cancer

**DOI:** 10.1186/s12957-016-0924-3

**Published:** 2016-06-28

**Authors:** Lin Yao, Kunlin Yang, Xuesong Li, Zheng Zhang, Cuijian Zhang, Kan Gong, Zhijun Xi, Zhisong He, Liqun Zhou

**Affiliations:** Department of Urology, Peking University First Hospital, No. 8 Xishiku St, Xicheng District, Beijing, 100034 China; Institute of Urology, Peking University, No. 8 Xishiku St, Xicheng District, Beijing, 100034 China; National Urological Cancer Center, No. 8 Xishiku St, Xicheng District, Beijing, 100034 China

**Keywords:** Retroperitoneal, Retroperitoneoscopic nephroureterectomy, Upper tract urothelial cancer

## Abstract

**Background:**

To evaluate the safety and efficacy of the completely retroperitoneoscopic nephroureterectomy (CRNU), a retrospectively comparative study between completely and traditionally retroperitoneoscopic nephroureterectomy (TRNU) was done in a single center.

**Methods:**

From January 2014 to December 2014, 107 patients with upper tract urothelial cancer (UTUC) underwent CRNU. The kidney was retroperitoneoscopically dissected and the bladder cuff was cut by endoscopic gastrointestinal automatic stapler, and the specimen was removed from a 6-cm incision by posterior axillary line. Demographic, perioperative, and follow-up data were collected and compared retrospectively with 110 patients undergoing TRNU.

**Results:**

The patients’ characteristics between the two groups were not statistically different (*p* > 0.05), and all patients successfully received the procedure. The mean operative time (106 ± 37.9 versus 199 ± 69.1 min, *p* < 0.0001), the mean estimated blood loss (47.2 ± 82.4 versus 166.9 ± 250.9 ml, *p* = 0.002), and the mean hospital stay (6.1 ± 3.5 versus 8.1 ± 3.3 days, *p* = 0.03) of the CRNU group decreased significantly compared to the traditional group. The operative time was not affected by gender. No open conversion and major complications occurred. The surgical margin of the ureter was all negative. The mean follow-up time was 13.4 months for the CRNU group and 37.5 months for the TRNU group. All follow-up patients in the CRNU group were alive without local recurrence. No cases of port site metastasis and local recurrence were observed in both groups. Bladder tumor recurrence occurred in 4 patients of the CRNU group and 21 patients of the TRNU group.

**Conclusions:**

The CRNU using an endoscopic gastrointestinal automatic stapler to manage the bladder cuff is feasible and advantageous in decreasing the operative time, the blood loss, and the hospital stay. However, a larger sample and longer follow-up time will be still required.

## Background

The golden standard surgical treatment for non-metastatic upper tract urothelial carcinoma (UTUC) is open radical nephroureterectomy with resection of the distal ureter and bladder cuff [1]. Since Clayman et al. firstly described the laparoscopic nephroureterectomy, it has been confirmed to be oncologically equally effective compared to open radical nephroureterectomy [2].

In our center, combining the retroperitoneoscopic nephrectomy with open distal ureterectomy with a bladder cuff has been performed to minimize the surgical mortality. In this traditional way, the patient’s position has to be changed from lateral position to supine position during the surgery while re-disinfection of open incision is also needed. Since January 2014, in order to improve the surgical procedure, we have developed a new surgical procedure defined as completely retroperitoneoscopic resection of the kidney and distal ureter with bladder cuff. We presented our initial evaluation of the safety and the efficacy of this procedure compared to the traditionally surgical procedure.

## Methods

From January 2014 to December 2014, 46 males and 61 females with UTUC of the renal pelvis and/or ureter received the completely retroperitoneoscopic nephroureterectomy (CRNU) performed by the same surgical team. The patients were preoperatively diagnosed by computerized tomography (positive rate, 103/107, 96.3 %), fluorescence in situ hybridization (positive rate, 92/107, 86.0 %), the urinary cytology (positive rate, 63/107, 58.9 %), or ureteroscopy (positive rate, 20/20). In these examinations, the ureteroscopic biopsy was not the essential examination, unless it was used for diagnostic uncertainty and the patient’s strongly requirement.

The data were retrospectively collected, including demographic data (age and gender), body mass index (BMI), the overall operative time (from the beginning to the end of the whole surgery, including the time of re-position, re-disinfection, and changing television monitor), the intraoperative blood loss, complications, open conversion rate, hospitalization stay, the removal time of the catheter and the drainage, the pathological outcome, the follow-up time, and the follow-up results. Data of 110 patients received traditionally retroperitoneoscopic nephroureterectomy (TRNU) from March 2010 to June 2014 operated by the same surgical team were also collected for comparisons.

Informed contents were accepted and signed off by all patients and their family members before surgery. The study was approved by the institutional review board from Peking University First Hospital.

### Surgical technique

All patients in the CRNU group successfully received the surgery. After the induction of general anesthesia, a transurethral catheter was inserted. The patient was placed in a side appropriate lateral decubitus position, and the waist was raised by placing a square mat under the waist.

**Fig. 1:**
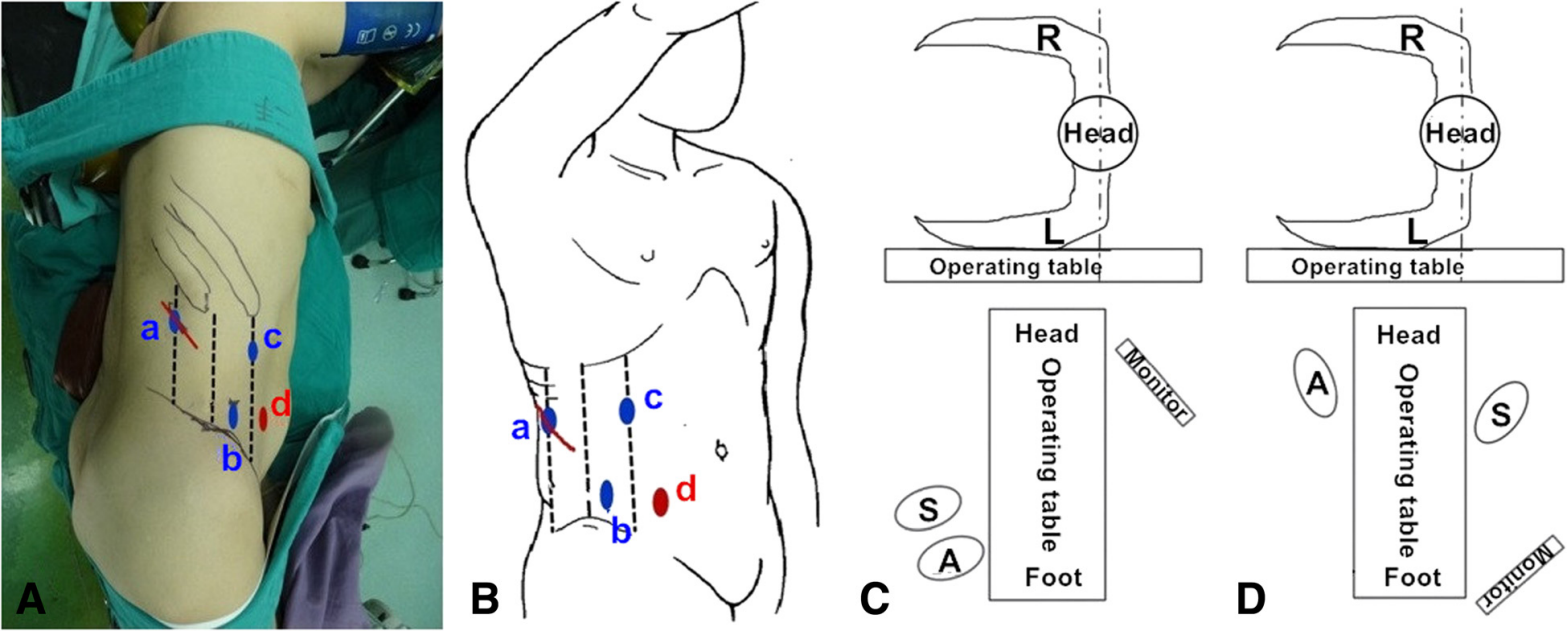
Trocar distribution and change of operative position (affected side is on the *right* in this example). **a**, **b** Point a: 2 cm below the crossing point of the posterior axillary line and the 12th rib margin. Point b: 3 cm ventrally to the crossing point of the midaxillary line and iliac crest. Point c: 2 cm below the costal margin in the anterior axillary line. Point d: at the McBurney’s point. **c**, **d** The positions of the television monitor, surgeon, and assistant when nephrectomy with mobilization of the proximal ureter and when distal ureterectomy with bladder cuff excision. *R* right, *L* left, S surgeon, *A* assistant

A 3-cm incision was made 2 cm below the crossing point of the posterior axillary line and the 12th rib margin (point a, see Fig. 1a, b). The muscle was bluntly dissected from the incision by a forceps until the extraperitoneal fat could be seen. The retroperitoneal space was expanded by the surgeon’s forefinger. A balloon was made from a size 8 surgical glove and tied to a 50-ml injector. Then, the homemade balloon was placed into the retroperitoneal space and inflated with 600 to 800 ml of air to get an appropriate working space. Guided by the surgeon’s forefinger, two 12-mm trocars were inserted at point b and point c (Fig. 1a, b). The trocar at point b was used as the camera port. Then, a 12-mm trocar was placed from the 3-cm incision which was sutured partially to fix the trocar.

**Fig. 2:**
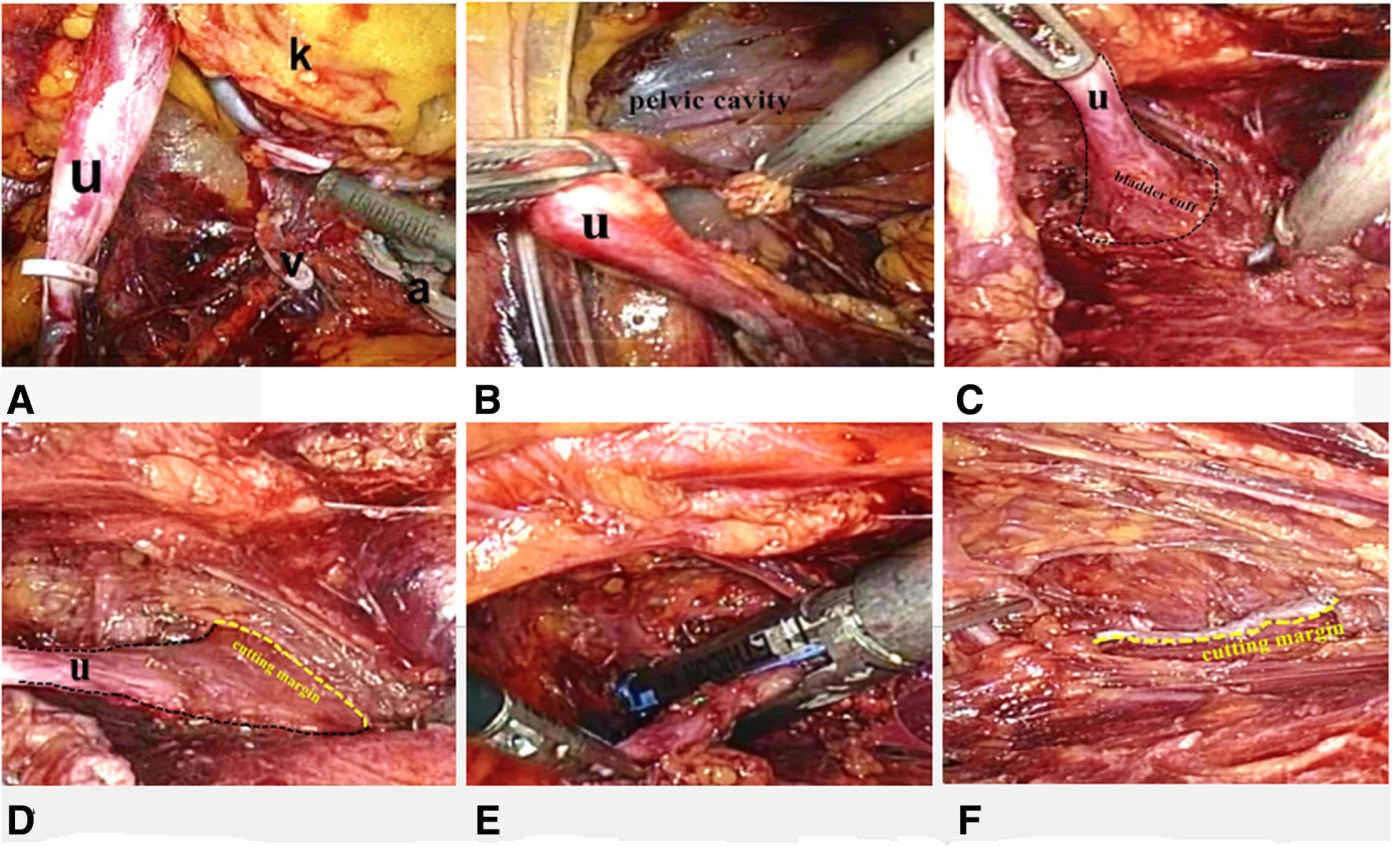
Operative view of main surgical steps. *u* ureter, *k* kidney, *v* renal vein, *a* renal artery. **a** Nephrectomy and mobilization of proximal ureter. The ureter is clamped by Hem-o-lok. **b** Distal ureter is dissected in the pelvic cavity. **c** Distal ureter with the bladder cuff marked with *black dotted line*. **d** The cutting margin of the required bladder cuff marked with *yellow dotted line*. **e** The bladder cuff is cut by endoscopic gastrointestinal automatic stapler. **f** The cutting margin of the required bladder cuff marked with *yellow dotted line*

Gerota’s fascia was opened after the extraperitoneal fat was dissected, and the dorsal side of the kidney was exposed outside the perirenal fat capsule. The upper segment of the ureter was dissected and clamped by Hem-o-lok. Then, the kidney was retroperitoneally dissected, including the renal vein, renal artery, the upper pole, the lower pole, and the ventral side of the kidney (Fig. 2a). The adrenal gland was reserved.

After, the ureter was dissected to the level of the iliac crest. The positions of the television monitor, surgeon, and assistant were changed (Fig. 1c, d). One more trocar was placed at McBurney’s point (point d, see Fig. 1a, b) if the affected side was on the right side (or the symmetrical equivalent to McBurney’s point for the tumor on the left side). The camera port was changed from point b to point c. Two trocars at point b and point d were used as operative ports. The ureter should be continuously dissected until to the vesicoureteral junction (Fig. 2b). For the female patient, uterine artery was cut after clipped by Hem-o-lok. The detrusor muscle was incised along the ureter in different directions while pulling the proximal ureter to obtain a 3-cm-wide bladder cuff around the ureteral orifice (Fig. 2c, d). Then, the distal ureter with bladder cuff was transected by a flexible endoscopic gastrointestinal automatic stapler (Endo-GIA) (Fig. 2e, f). Lymphadenectomy could be performed if needed. The specimen was put into an endoscopic specimen bag. The incision of the port on the posterior axillary line was extended to be a 6-cm incision to remove the specimen. At that time, the specimen must be confirmed to contain the required bladder cuff by the surgeon. After the confirmation of the complete removal of the bladder cuff, a F20 drain was placed into the pelvic cavity through port b. Sometimes, an additional drain tube was put in the renal bed. At last, the incision and the port sites without drainages were closed and the drainages were fixed. The patient-controlled analgesia pumps are routinely used after surgery if there is no contraindication.

**Fig. 3:**
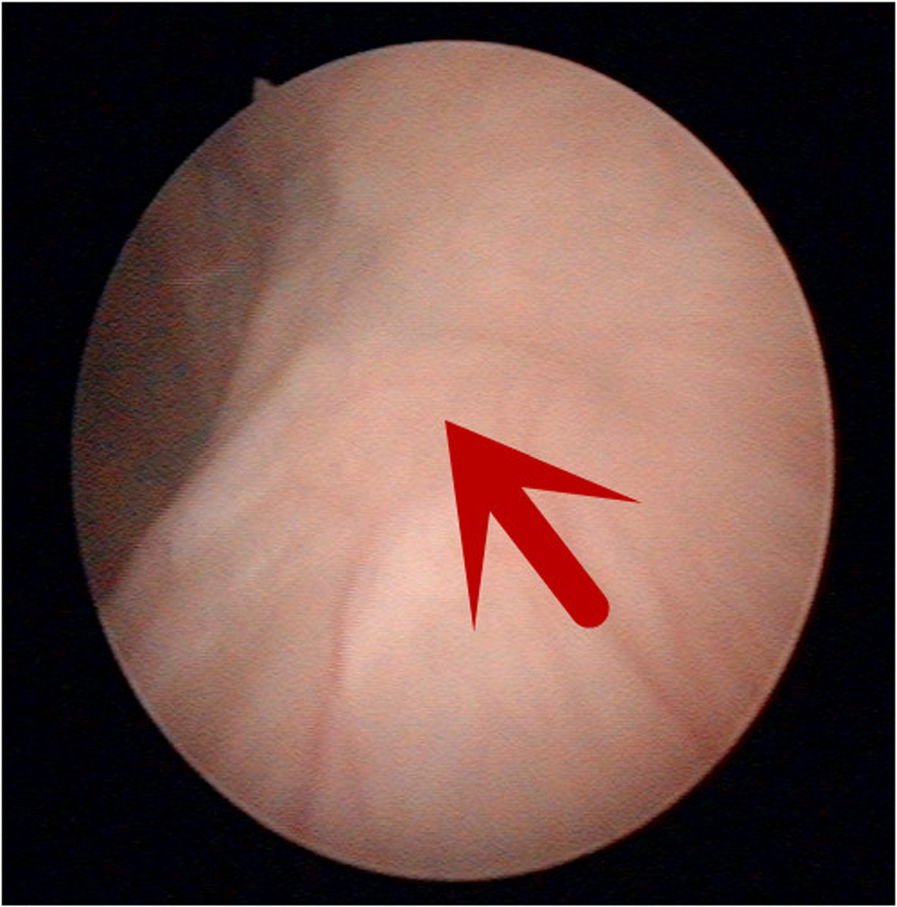
The healed bladder incision marked with the *red arrow* is no ureteral orifice on the affected side when under cystoscopy examination 3 months after surgery

Ninety-five patients received single bladder instillation of the pirarubicin (dosage, 40 mg) or the epirubicin (dosage, 50 mg) within 48 h after surgery. No bladder leakage occurred. All patients received cystoscopic observation 3 months after surgery, and no stone formation was seen in the bladder. The cystoscopy observation also showed that the bladder incision healed well, and no ureteral orifice was found on the affected side (Fig. 3). Further follow-up consisted of cystoscopy, urinary cytology every 3 to 6 months, and computed tomography every 6 months.

Lymphadenectomy is still not routinely performed at RNU. Only three patients of invasive UTUC in the CRNU group whose preoperative image indicated the suspicious enlarged lymph nodes received regional lymphadenectomy. No patient in the TRNU group received lymphadenectomy.

Statistical analysis was performed using Mann–Whitney *U* test and Student *t* test to evaluate statistical differences between different groups. Difference was considered statistically significant if *p* value was <0.05.

## Results

The comparisons of patient’s characteristics between CRNU and TRNU are shown in Table 1, and no significant differences are detected in gender, age, BMI, and operative side (*p* > 0.05, Table 1).

**Table 1:** Patient’s characteristics, surgical results, and follow-up outcomes of CRNU and TRNU

Parameter	CRNU	TRNU	*p* value
No. patients, *n*	107	110	
Sex			0.91
Male	46	48	
Female	61	62	
Median age, year (range)	71 (55–82)	71 (52–88)	0.51
Mean BMI, kg/m^2^ (range; ±SD)	23.2 (18.1–28.1; ±2.8)	23.5 (17.6–29; ±2.9)	0.50
Operative side, *n*			0.71
Left	52	51	
Right	55	59	
Tumor location			
Renal pelvis	62	68	
Ureter	39	37	
Upper	21	18	
Mid	4	10	
Distal	14	9	
Multiple location	6	5	
Mean overall operating time, min (range; ±SD)	106.0 (46–216; ±37.9)	199.5 (100–416; ±69.1)	<0.0001
Male	105.0 (76–172; ±25.7)	218.3 (128–416; ±71.9)	<0.0001
*p* value (Male versus Female)	0.93 > 0.05	0.06 > 0.05	
Female	106.5 (46–216; ±42.3)	185.1 (100–410; ±63.2)	<0.0001
Mean estimated blood loss, ml (range; ±SD)	47.2 (5–400; ±82.4)	166.9 (20–1450; ±250.9)	0.002
RNU with lymph node dissection			
No. of patients	3	No	
Open conversion	No	No	
Intraoperative and postoperative complications			
(n/Clavien Classification)			
UTI	4/grade II	7/grade II	
Lymphatic leakage	4/grade I	2/grade I	
Others	0	0	
Single bladder instillation of chemotherapy	95	97	
The removal time of drain after surgery, days (range; ± SD)	4.0 (2–17; ±3.0)	4.2 (2–12; ±1.5)	0.79
Mean hospital stay, days (range; ±SD)	6.1 (3–21; ±3.5)	8.1 (4–26; ±3.3)	0.03
The removal time of urethral catheter after surgery, week	1	1	
Pathologic stage, *n*			
pTaN0M0	8	6	
pT1N0M0	63	65	
pT1N1M0	0	1	
pT2N0M0	30	23	
pT2N2M0	1	0	
pT3N0M0	4	14	
pT3N1M0	1	0	
pT4N2M0	0	1	
Pathologic grade (G), *n*			
G1	38	31	
G2	54	52	
G3	15	27	
The surgical margin of the distal ureter (negative or positive)	Negative	Negative	
Mean follow-up time, month (range; ±SD)	13.4 (6–18; ±2.1)	37.5 (13–63; ±15.9)	
No. of patients of follow-up (*n*)	105	106	
Bladder tumor recurrence			
No. patients of follow-up (*n*)	4	21	

*BMI* body mass index, *CRNU* completely retroperitoneoscopic nephroureterectomy, *TRNU* traditionally retroperitoneoscopic nephroureterectomy, *UTI* urinary tract infection

The surgical outcomes of the two groups are also shown in Table 1. The mean overall operative time (106.0 ± 37.9 versus 199.5 ± 69.1 min, *p* < 0.0001), the mean estimated blood loss (47.2 ± 82.4 versus 166.9 ± 250.9 ml, *p* = 0.002), the mean hospital stay (6.1 ± 3.5 versus 8.1 ± 3.3 days, *p* = 0.03) of the CRNU group were significantly reduced compared to TRNU while the overall operative time was not affected by gender (*p* > 0.05, Table 1), and no open conversion occurred. No major complications were observed in both groups except four patients in the CRNU group, and seven patients in the TRNU group suffered urinary tract infection and been cured with antibiotics for 1 week. Lymphatic leakage occurred in four patients of the CRNU group and two patients of the TRNU group.

The pathological diagnoses are shown in Table 1. The surgical margin of the ureter was all negative. In three patients who received lymphadenectomy, two patients had positive lymph nodes and one patient had negative result. Two patients in the CRNU group and four patients in the TRNU group were lost to follow-up because we could not connect with them. The mean follow-up time was 13.4 ± 2.1 months in the CRNU group and 37.5 ± 15.9 months in the TRNU group. All follow-up patients in the CRNU group were alive without local recurrence. No cases of port site metastasis and local recurrence were observed in both groups. Bladder tumor recurrence occurred in four patients in the CRNU group treated with transurethral resection and in 21 patients in the TRNU group.

## Discussion

Although the golden standard treatment for localized high-risk UTUC is still the radical nephroureterectomy with excision of the bladder cuff [1], with extensive progress of minimally invasive techniques, the laparoscopic nephroureterectomy (LNU) has evolved into an effective alternative to open nephroureterectomy (ONU) [3]. More and more studies have supported that LNU has a comparable oncological outcomes to ONU. In addition, LNU can reduce the blood loss, hospital stay, and post-operative pain compared with ONU [4–9]. In this study, the mean operative time, the mean estimated blood loss, and the mean hospital stay in the CRNU group are further decreased compared to the TRNU group.

The radical nephroureterectomy can be divided into two main steps: nephrectomy with mobilization of the proximal ureter and distal ureterectomy with bladder cuff excision. In our center, traditionally, the nephrectomy and the mobilization of the proximal ureter are performed by retroperitoneal approach, but the distal ureterectomy with bladder cuff is performed by open approach. The patient’s position needs to be changed from lateral position to supine position, and one more disinfection of the incision should be performed. To facilitate this process and reduce the operative time, we tried to perform a completely retroperitoneal approach with no need to change the patient’s position. By this way, we just take about 3 to 5 min to adjust during the midway surgery and the operative time dramatically decreases.

The management of distal ureter is a very important step in either laparoscopic procedure or open procedure. It may be an independent predictor of oncological outcome. In 2014, Krabbe et al. reported that the surgical management of the distal ureter without excision of a transvesical bladder cuff resulted in significantly worse non-intravesical recurrence (IVR)-free survival and cancer-specific survival but had no influence on IVR [10]. This study moved us to standardize the resection of bladder cuff.

There have been many approaches to manage the bladder cuff, including transvesical, extravesical, and endoscopic procedures [3, 4, 11–14]. Among these methods, no differences in non-bladder recurrence and survival exist; however, the endoscopic approach has a higher rate of intravesical recurrence [11].

In 1995, McDougall et al. firstly reported the extravesical transection of the distal ureter with the bladder cuff using an Endo-GIA which is an extravesical approach [4]. Then, Yoshino et al. reported this method to manage the bladder cuff by completely retroperitoneal approach in 2003 [14]. They concluded that the Endo-GIA was an efficient and low-risk procedure. Both our technique and Yoshino’s technique are the completely retroperitoneal approach. But the number of the ports (four ports in our technique and five ports in Yoshino’s technique) and the procedures to dissect the distal ureter are different. As the complete retroperitoneal approach does not enter into the abdominal cavity, the abdominal cavity implantation metastasis can be avoided maximally. But the biggest limitation of these studies and this study was the lack of long-term follow-up.

In addition, another disadvantage was that the complete removal of the bladder cuff could not be confirmed using the Endo-GIA [14]. So, we must examine whether the dissected specimen contains the required bladder cuff immediately after the specimen is taken out as a urethral catheter is inserted before surgery to drain out the urine to decrease the bladder filling pressure and to avoid the urine extravasation as much as possible when the bladder cuff is cut by the Endo-GIA.

Because the urothelium between the staples of Endo-GIA remains after transection, the incomplete excision of bladder cuff can lead to a high recurrence rate [15], and the remaining urothelium may be a source of recurrence. So the use of staples is no longer considered as effective technique for complete and safe bladder cuff removal in the ICUD-SIU consultation for the treatment of localized high-risk UTUC updated by 2016 [16]. However, the traditional method of closing the bladder wall with sutures also faces the risk of 3 to 5 mm of the remaining urothelium [14].

The staples may be exposed inside the bladder and lead to stone formation in the long run. However, we did not found stone formation and the staples under cystoscopy during follow-up time. Chandhoke et al. also found neither staples nor stone formation [17].

Generally, surgery on male patients might be more difficult as the female pelvis is wider than the male’s. However, in our study, there was no significant statistical difference (*p* > 0.05) on the operative time between the male and the female.

It is reported that 40 % of patients after nephroureterectomy will develop bladder recurrence potentially derived from implantation of the primary tumor. O’Brien et al. reported that a single post-operative dose of intravesical mitomycin C could reduce the risk of a bladder tumor within the first year after nephroureterectomy [18]. Another prospective randomized phase II by Ito et al. also reported that the patient who received a single early intravesical instillation of pirarubicin had a lower bladder recurrence rate than the non-instillation group at 1 year (16.9 % versus 31.8 %) and 2 years (16.9 % versus 42.2 %) [19]. In the European Association of Urology guidelines on UTUC updated by 2015 and the ICUD‑SIU consultation on localized high-risk UTUC updated by 2016, post-operative instillation of chemotherapy is recommended to avoid bladder recurrence [1, 16]. In our center, the patients are generally recommended a single post-operative intravesical instillation of pirarubicin or epirubicin within 48 h after surgery.

## Conclusions

In conclusion, the CRNU using an Endo-GIA to cut the bladder cuff is advantageous in decreasing operative time, the blood loss, and the hospital stay. However, a larger sample, longer follow-up time, and further analysis are still needed to confirm its oncological outcome.

## Abbreviations

BMI, body mass index; CRNU, completely retroperitoneoscopic nephroureterectomy; Endo-GIA, endoscopic gastrointestinal automatic stapler; IVR, intravesical recurrence; LNU, laparoscopic nephroureterectomy; ONU, open nephroureterectomy; TRNU, traditionally retroperitoneoscopic nephroureterectomy; UTUC, upper tract urothelial carcinoma
